# Supplementary material for: C. elegans orthologs MUT-7/CeWRN-1 of Werner syndrome protein regulate neuronal plasticity
Source: eLife. 2021 Mar 1;10:e62449. doi: 10.7554/eLife.62449 (PMC7946423; doi:10.7554/eLife.62449)
Supplement: Supplementary file 1. — The cell fate of the AWC neuron was examined by quantifying the asymmetric expression of pstr-2::DsRed. Animals were scored in three categories according to pstr-2::DsRed expression either in AWC (0AWCpstr-2 ON), in only one AWC (1AWCpstr-2 ON), or in both AWCs (2AWCpstr-2 ON). [file elife-62449-supp1.docx]

Supplementary File 1

| Genotype | Percentage of animals with | | | |
| --- | --- | --- | --- | --- |
|  | 0AWCp*str-2* ON | 1AWCp*str-2* ON | 2AWCp*str-2* ON | n |
| *WT* | 0 | 100 | 0 | 83 |
| *Cewrn-1(gk99)* | 0 | 100 | 0 | 69 |
| *mut-7(pk204)* | 0 | 100 | 0 | 71 |
| *mut-7(pk204)* ex[CterGFP-MUT-7] | 0 | 100 | 0 | 82 |
| *mut-7(pk204)* ex[NLS-GFP-MUT-7] | 0 | 96.8 | 3.2 | 31 |
