# Supplementary material for: C. elegans orthologs MUT-7/CeWRN-1 of Werner syndrome protein regulate neuronal plasticity
Source: eLife. 2021 Mar 1;10:e62449. doi: 10.7554/eLife.62449 (PMC7946423; doi:10.7554/eLife.62449)
Supplement: Supplementary file 2. — Integrated GFP-tagged EGL-4 (termed pyIs500) was expressed in wild-type, hpl-2(tm1489), and Cewrn-1(gk99) animals. The nuclear expression of EGL-4 was scored in naïve and odor-trained animals. [file elife-62449-supp2.docx]

Supplementary File 2

| Genotype | Nuclear EGL-4 in the AWC nucleus (%) | |
| --- | --- | --- |
|  | Naïve animals | Odor-trained animals |
| *N2*; pyIs500 | 3.8% (n=130) | 98.3% (n=120) |
| *hpl-2*; pyIs500 | 3.5% (n=113) | 97.5% (n=79) |
| *Cewrn-1*: pyIs500 | 3.6% (n=139) | 97.5% (n=80) |
